# Using bookmarking methods with orthopedic clinicians and patients with fractures produces score interpretation labels for patient-reported outcome measures

**DOI:** 10.1007/s11136-023-03439-5

**Published:** 2023-05-25

**Authors:** Nan E. Rothrock, Sandra A. Wilson, Marilyn Heng, Aleksandra Hodor, Alexander Joeris, Aaron J. Kaat, Karma McKelvey, Benjamin D. Schalet, Mark Vrahas

**Affiliations:** 1 Feinberg School of Medicine of Northwestern University, Chicago, IL USA; 2 Rowan University School of Osteopathic Medicine, Stratford, NJ USA; 3 Department of Orthopaedics, University of Miami Miller School of Medicine, Miami, FL USA; 4 Orthopaedic Trauma Service, Ryder Trauma Center, Jackson Memorial Hospital, Miami, FL USA; 5 AO Innovation Translation Center, AO Foundation, Dubendorf, Switzerland; 6 Department of Orthopaedics, Cedars-Sinai Medical Center, Los Angeles, CA USA; 7 Department of Medical Social Sciences, Northwestern University Feinberg School of Medicine, 625 N Michigan Ave, Suite 2700, Chicago, IL 60611 USA

**Keywords:** Patient-reported outcome measure, Orthopedics, Fractures, PROMIS, Physical function, Pain interference, Score thresholds

## Abstract

**Objective:**

The objective of this study was to determine the patient-reported outcome measure (PROM) score ranges associated with descriptive labels (i.e., within normal limits, mild, moderate, severe) by using bookmarking methods with orthopedic clinicians and patients who have experienced a bone fracture.

**Study design and setting:**

We created vignettes comprised of six items and responses from the Patient-Reported Outcomes Measurement Information System (PROMIS) Upper Extremity Function, Physical Function, and Pain Interference item banks reflecting different levels of severity. Two groups of patients with fractures (*n* = 11) and two groups of orthopedic clinicians (*n* = 16) reviewed the vignettes and assigned descriptive labels independently and then discussed as a group until reaching consensus via a videoconference platform.

**Results:**

PROMIS Physical Function and Pain Interference thresholds (*T* = 50, 40, 25/30 and *T* = 50/55, 60, 65/70, respectively) for patients with bone fractures were consistent with the results from other patient populations. Upper Extremity thresholds were about 10 points (1 SD) more severe (*T* = 40, 30, 25/20) compared to the other measures. Patient and clinician perspectives were similar.

**Conclusion:**

Bookmarking methods generated meaningful score thresholds for PROMIS measures. These thresholds between severity categories varied by domain. Threshold values for severity represent important supplemental information to interpret PROMIS scores clinically.

## Introduction

Restoring physical function while minimizing pain is the primary aim of the orthopedic care. Consequently, patient-reported outcome measures (PROMs) for these concepts have been widely utilized in clinical research to understand the impact of an injury or condition and the impact of an intervention [1, 2]. PROMs are increasingly integrated into routine clinical practice to inform individual patient care [3, 4]. More recently, PROMs are being used in the evaluation of healthcare quality [5, 6]. In order to understand and use data gathered for these distinct reasons, PROM scores need to be interpretable [7].

Score interpretability has been cited as one of the challenges in implementing PROMs in clinical settings [8–11]. In most cases, a PROM’s metric provides some information. For example, measures that produce 0–100 scores enable a PROM user to identify how a given patient compares relative to the absolute minimum and maximum possible. A standard score (e.g., *T*-score) allows a user to identify how a patient compares to the mean of a known group using the standard deviation (SD) as a unit of measure. For example, the Patient-Reported Outcomes Measurement Information System (PROMIS) measures include item banks for the assessment of many patient-reported outcome domains using a *T*-score with a general population mean of 50 and SD of 10. However, knowing a patient is 1 SD above average does not provide information about how this level of severity manifests in a patient’s day-to-day life, nor does it provide information sufficient to inform a clinical action. Tools to translate numeric scores into interpretable, descriptive labels are needed.

One approach to understanding different score ranges is “bookmarking” [12]. Bookmarking is a standard setting methodology used in educational testing to identify the meaningful thresholds on a test (e.g., pass/fail point in a licensing exam). In this context, validated test questions are presented to experts (e.g., curriculum developers, instructors) ordered by level of difficulty (i.e., easiest to hardest). Experts review test questions and “bookmark” the location between the last test question a person at one level of proficiency (e.g., proficient) would be expected to answer correctly and the next question they may not be able to answer correctly. Hence, it reflects where the next level of proficiency begins (e.g., advanced). Experts convene to discuss bookmark placements before making final decisions. This approach has been adapted for PROMs [13] and applied in oncology [14, 15], neurology [16], spinal cord injury [17], and rheumatology [18–21] patient populations. Here, instead of test questions, PROM items with responses reflecting hypothetical patients are used. These “vignettes” of patients reporting different levels of severity or impairment are reviewed by patients and clinicians and assigned labels (e.g., mild, moderate, severe). This creates meaningful thresholds between levels of severity (e.g., transition point from mild to moderate) that can facilitate PROM score interpretation.

Our study aimed to apply bookmarking methods in a videoconference platform to create meaningful labels for ranges of PROM scores for Upper Extremity Function, Physical Function, and Pain Interference in patients who have experienced a bone fracture.

## Materials and methods

### Measures

Items included in vignettes of hypothetical patients were selected from three PROMIS item banks in English. All PROMIS measures are scored as *T*-scores, a standard score with a mean of 50 in the US general population and SD of 10. Item banks assessed outcomes central to orthopedic care: physical function and pain.

PROMIS Bank v2.1—Upper Extremity (Upper Extremity Function) includes 46 items measuring capability to perform activities that require the use of shoulders, arms, and hands [22]. Higher scores indicate better upper extremity function.

PROMIS Bank v2.0—Physical Function (Physical Function) assesses capability to perform activities that use one’s upper extremities (e.g., opening containers), lower extremities (e.g., walking), neck and back as well as instrumental activities of daily living (e.g., dressing) [23]. The bank includes 165 items and higher scores indicate better function.

PROMIS Bank v1.1—Pain Interference (Pain Interference) includes 40 items assessing the degree to which pain interferes with engagement in social, cognitive, emotional, physical, and recreational activities [24]. Higher scores indicate more problems from pain.

### Procedures

#### Vignette construction

**Fig. 1:**
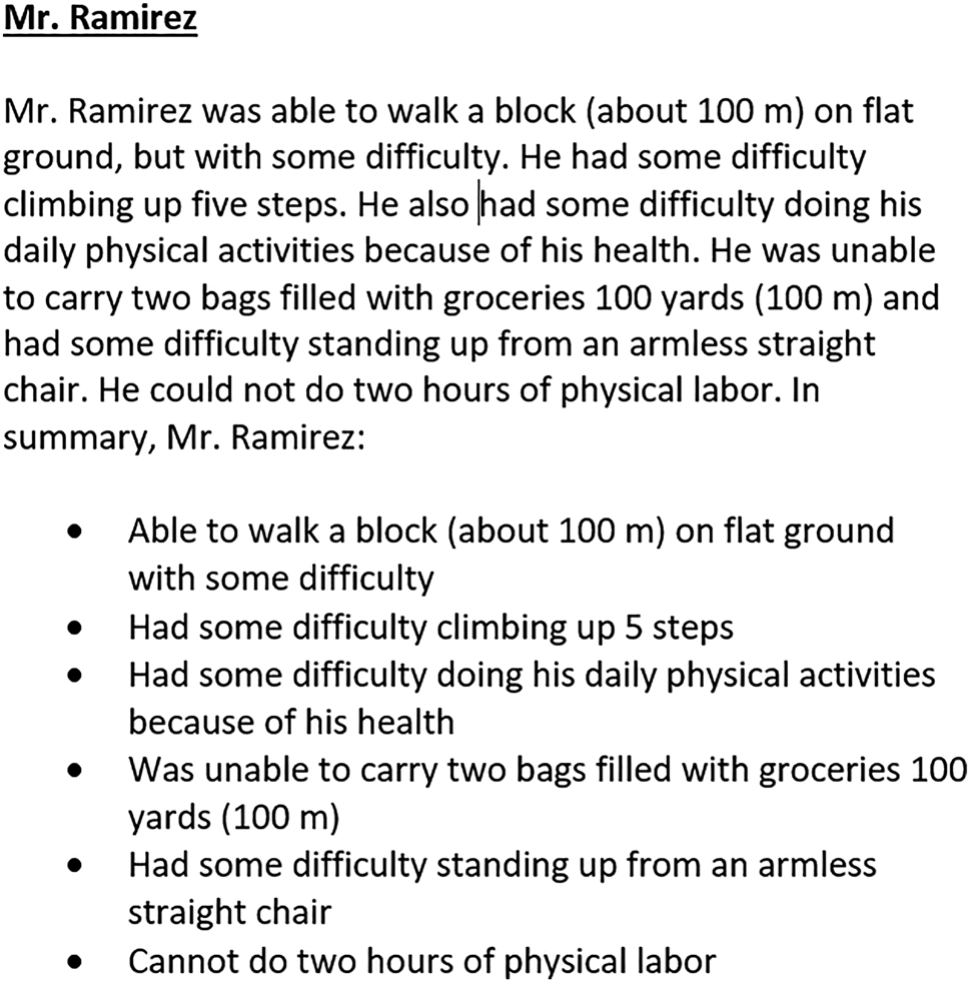
Vignette for Physical Function *T*-score = 32.5

We constructed vignettes based on three PROMIS item banks [13]. Item banks were previously calibrated using an item response theory model, which allows for any combination of items to produce a *T*-score on the same metric. Briefly, to create vignettes of hypothetical patients, we calculated the most likely response to every item in each item bank for all possible *T*-scores. For example, a person with very poor physical function (e.g., *T* = 30) would most likely respond “unable to do” to an item about one’s ability to climb up five steps whereas a high functioning person (e.g., *T* = 60) would most likely respond “without any difficulty.” We then identified the lowest and highest *T*-scores that had variability in the most probable response options across items. This range varied by item bank. Vignettes were constructed at 5-point intervals within that range. For Upper Extremity Function, the lowest score selected for a vignette was 17.5. Seven additional vignettes (22.5, 27.5, 32.5, 37.5, 42.5, and 47.5) were then created by selecting items with their most likely response at a given *T*-score. For Physical Function, nine vignettes were created for *T*-scores between 17.5 and 57.5. For Pain Interference, seven vignettes were constructed for *T*-scores between 47.5 and 77.5. Each vignette included six items with only one common item across vignettes for a given domain. Items were selected to maximize variation in response options within a vignette and represent the range of content within an item bank. Upper Extremity Function and Pain Interference vignettes were created de novo and included a total of 33 and 32 items, respectively, across all vignettes. Physical Function vignettes were modified from vignettes used in a previous study [14]. First, to minimize conceptual overlap between the vignettes for Physical Function and Upper Extremity Function, items in the Physical Function vignettes that focused on upper extremity activities were removed and replaced with non-upper extremity items. Second, we added items reflecting better physical function that had been added to the PROMIS Physical Function item bank [23] after the original bookmarking study. A total of 44 items were used. Each vignette’s hypothetical patient was named with a common surname based on the 2010 US Census (see Fig. 1). We pilot tested the vignettes for all three domains to assess how well they reflected distinct levels of function/pain by asking blinded team members and colleagues to order vignettes from least to most severe.

#### Patient participants

A study coordinator screened sequential patients receiving orthopedic care at an academic medical center in the Northeastern US for eligibility. Eligibility criteria included the following: fracture of the hip, proximal humerus, distal radius, tibial shaft, ankle, or wrist confirmed radiographically or by an attending physician in the past year, age over 18 years old, proficient with oral and written English, ability to send and receive email, and ability to join a focus group from a laptop or desktop computer using a videoconference platform. In order to include diverse perspectives, each group consisted of at least one male and one female, at least 1 person with an injury 1–3 months ago, and at least 2 Latinx, Black, Indigenous, or person of color participants. The study coordinator then approached eligible patients, confirmed eligibility, and provided additional information about the study. Enrolled participants received study materials (e.g., paper copies of vignettes and bookmarks) and were scheduled for a video call in advance of the focus group. During the video call, the study coordinator provided instruction, if needed, on how to use the platform, tested their connectivity, answered questions about the study, and collected sociodemographic information.

Of the 118 patients screened, 86 met clinical eligibility criteria, and 58 were approached. Reasons for not approaching potential participants included the patient missing their appointment (5), the coordinator missing the patient due to schedule conflicts (10), and having already met targets for patients with similar sociodemographic and clinical characteristics (13). Eighteen patients (31%) agreed to participate. Of these, 11 attended one of two focus groups. Participants were compensated for their time.

#### Clinician participants

Clinician members of the study team emailed study information and an invitation to participate to institutional listservs comprised of clinicians serving patients with fractures (e.g., departments of physical therapy, occupational therapy, orthopedics, physical medicine, and rehabilitation; orthopedic advanced practice providers). Additional invitations were distributed to a regional orthopedic society and clinicians in their personal networks. Targeted participants encompassed both the West Coast and Northeast regions of the United States. The study coordinator contacted interested clinicians to complete an eligibility screen and collect sociodemographic information. Similar to the patient group, to include diverse perspectives, each group included a minimum of at least one surgeon and one non-surgeon, and at least one clinician from each region. Enrolled clinician participants were provided study materials in-person or via mail.

Forty-two clinicians expressed interest and were contacted to determine the eligibility. Seven did not complete the eligibility screening. Nine did not meet the eligibility criteria, including treating patients with fractures for more than 1 year post-training, ability to participate in a videoconference from a laptop or desktop computer, and availability at the time of the focus group. Ten responded to the request to complete the eligibility screening after enrollment targets were met. The 16 remaining clinicians were approached and all agreed to participate. All attended one of two focus groups. Clinician participants were compensated for their time and received continuing medical education credit.

#### Study protocol

All focus groups were three hours long and began with reviewing guidelines for using the videoconference platform including using cameras so all participants were visible. After an explanation of the aims and methods, participants practiced the methods in a warm-up exercise. Next, they were asked to define the severity labels “within normal limits,” “mild,” “moderate,” and “severe.” They were then instructed to complete a seven to ten item short form from the target item bank on paper. These were not collected but used to make participants more familiar with the types of items used to measure a domain and think more deeply about the concept being assessed. Using a paper document that displayed each vignette in order from least severe to most severe, participants placed bookmarks separating vignettes that reflected hypothetical patients who were “within normal limits” or who had “mild,” “moderate,” or “severe” dysfunction or interference. Participants worked independently and reported their results via REDCap, a web-based data collection platform, through a personalized URL sent via the videoconference platform’s messaging tool. Participants then engaged in a moderated discussion sharing how they described each vignette and how they arrived at their conclusion. The moderator displayed the vignettes in the videoconference platform and moved bookmarks in real time to reflect where participants suggested they should be placed. The moderator used a semi-structured guide with specific prompts (e.g., “How did you decide on the location of the bookmark?”, “Knowing that we want to reach an agreement, is anyone persuaded to move their bookmark?”). The discussion continued until there was consensus on bookmark placement. This study was approved by the Institutional Review Board at the lead study site.

### Data analysis

Individual participants’ initial bookmark placement was reviewed for illogical order (e.g., “mild” assigned to more severe vignettes than “moderate”). Next, for each domain, we compared the consensus for bookmark placement across the four groups. The threshold between categories (e.g., mild/moderate) was calculated as the midpoint between the vignettes on either side of a bookmark. For example, a bookmark between a vignette for *T* = 37.5 and a vignette for *T* = 42.5 equated a threshold of 40. When all four groups were not unanimous about a bookmark’s placement, we reviewed participants’ independent bookmark placement.

## Results

Of the 11 patient participants, the majority were female (*n* = 9, 82%), white (*n* = 8, 73%), partnered (*n* = 8, 73%), and working (*n* = 6, 55%; see Table 1). Mean age was 46 years old (SD = 18, range 25–71). All were highly educated. They had sustained a range of fractures (i.e., ankle, hip, tibia, wrist, multiple) on average 3.9 months earlier (SD = 3.0, range 1–10 months). Their treatments were mostly operative (*n* = 9, 82%; e.g., plate and screws, nails) and fractures were unhealed for all but one participant.

**Table 1:** Participant demographics

	Patient participants		Clinician participants	
	Number	Percentage	Number	Percentage
Sex				
Female	9	82%	9	56%
Male	2	18%	7	44%
Mean age	46 (SD = 18)	Range 25–71	41 (SD = 15)	Range 25–78
Race				
Asian	2	18%	3	19
Black or African American	1	9%	0	0%
White	8	73%	12	75%
Multiple	0	0%	1	6%
Spanish/Hispanic/Latino	1	9%	0	0%
Education				
College or vocational certificate	6	55%	0	0%
Post-graduate degree	5	45%	16	100%
Employment status				
Full-time employed	5	45%		
Part-time employed	1	9%		
Unemployed	2	18%		
Retired	3	27%		
Type of fracture				
Ankle	5	45%		
Hip	2	18%		
Tibial shaft	1	9%		
Wrist	2	18%		
Multiple	1	9%		
Months since injury	3.9 (SD = 3.0)	Range 1–10		
Profession				
Occupational Therapist			3	19%
Physiatrist			1	6%
Physical Therapist			6	38%
Physician Assistant			2	13%
Surgeon			4	25%
Mean years in orthopedic practice			13 (SD = 12)	Range 2–46

Of the 16 clinician participants, the majority were female (*n* = 9, 56%) and white (*n* = 12, 75%). Mean age was 41 years old (SD = 15, range 25–78) with on average 13 years of practice experience (SD = 12, range 2–46 years). Participants were physical therapists, surgeons, occupational therapists, physician assistants, and physiatrists.

One patient participant reported an illogical bookmark order placement for Upper Extremity Function and Pain Interference and was therefore removed from analyses. All other participants’ bookmark placement was logical (i.e., within normal limits, mild, moderate, and severe in order).

**Fig. 2:**
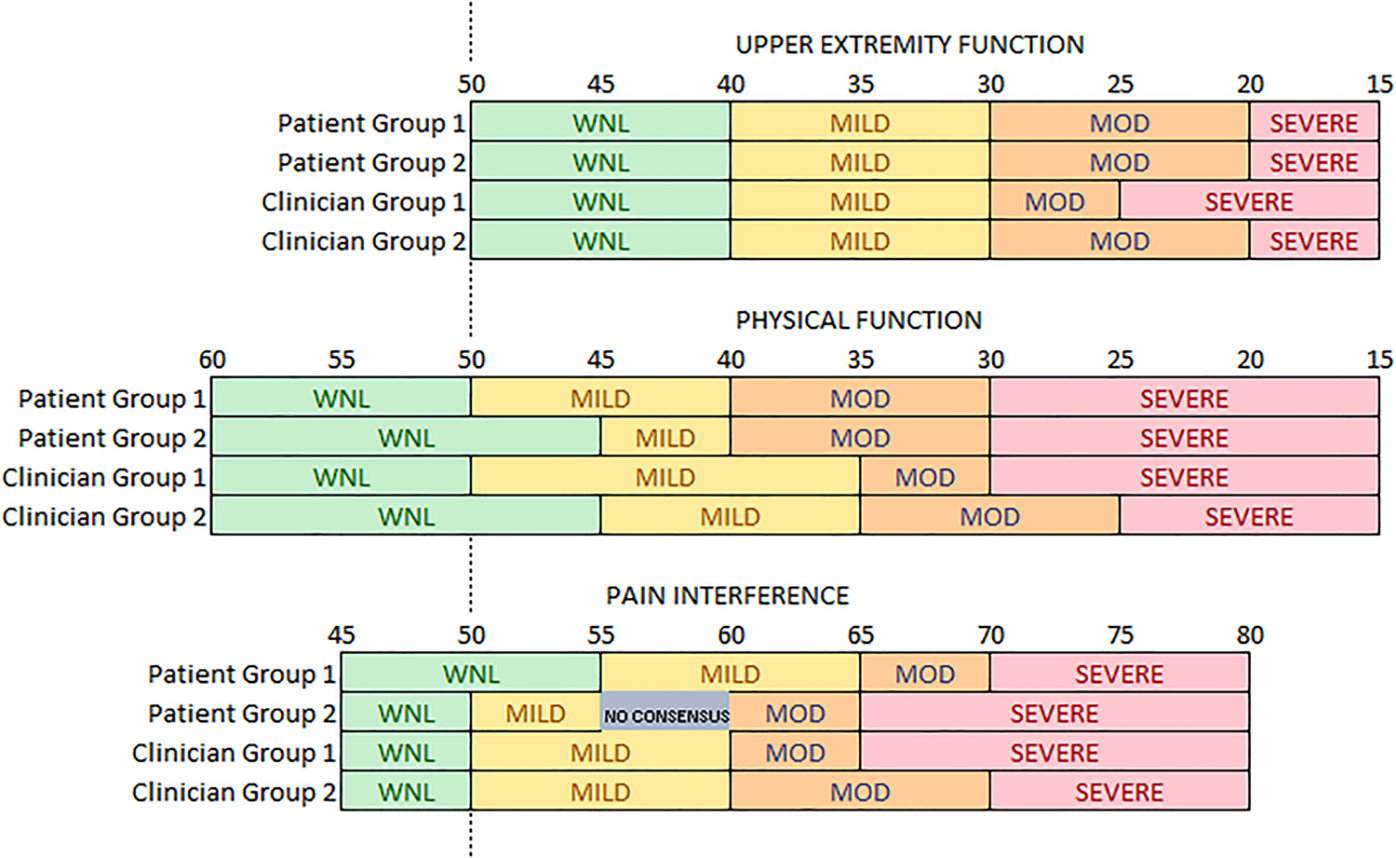
Patient and clinician groups’ consensus on thresholds between severity categories. *MOD* moderate, *WNL* within normal limits

For Upper Extremity Function, all four focus groups identified an identical thresholds between within normal limits and mild (*T* = 40) as well as between mild and moderate (*T* = 30, see Fig. 2). Although 3 of 4 groups identified *T* = 20 as the threshold between moderate and severe, when working independently prior to discussion, about half of all participants identified the threshold as *T* = 25 (see Table 2).

**Table 2:** Participants’ initial assignment of severity labels to vignettes

Upper Extremity Function *T*-score	47.5	42.5	37.5	32.5	27.5	22.5	17.5
Patients^a^							
WNL	10 (100%)	8 (80%)	1 (10%)	–	–	–	–
Mild	–	2 (20%)	9 (90%)	7 (70%)	1 (10%)	–	–
Moderate	–	–	–	3 (30%)	8 (80%)	6 (60%)	–
Severe	–	–	–	–	1 (10%)	4 (40%)	10 (100%)
Clinicians							
WNL	16 (100%)	12 (75%)	–	–	–	–	–
Mild	–	4 (25%)	16 (100%)	12 (75%)	3 (19%)	–	–
Moderate	–	–	–	4 (25%)	12 (75%)	7 (44%)	–
Severe	–	–	–	–	1 (6%)	9 (56%)	16 (100%)

Physical Function *T*-score	57.5	52.5	47.5	42.5	37.5	32.5	27.5	22.5	17.5
Patients									
WNL	11 (100%)	9 (82%)	4 (36%)	1 (9%)	–	–	–	–	–
Mild	–	2 (18%)	5 (45%)	7 (64%)	3 (27%)	–	–	–	–
Moderate	–	–	2 (18%)	3 (27%)	8 (73%)	9 (82%)	7 (64%)	1 (9%)	–
Severe	–	–	–	–	–	2 (18%)	4 (36%)	10 (91%)	11 (100%)
Clinicians									
WNL	16 (100%)	16 (100%)	5 (31%)	–	–	–	–	–	–
Mild	–	–	11 (69%)	16 (100%)	7 (44%)	–	–	–	–
Moderate	–	–	–	–	9 (56%)	16 (100%)	11 (69%)	–	–
Severe	–	–	–	–	–	–	5 (31%)	16 (100%)	16 (100%)

Pain Interference *T*-score	47.5	52.5	57.5	62.5	67.5	72.5	77.5
Patients^a^							
WNL	10 (100%)	7 (70%)	1 (10%)	–	–	–	–
Mild	–	3 (30%)	8 (80%)	4 (40%)	–	–	–
Moderate	–	–	1 (10%)	6 (60%)	5 (50%)	–	–
Severe	–	–	–	–	5 (50%)	10 (100%)	10 (100%)
Clinicians							
WNL	16 (100%)	6 (38%)	0 (0%)	–	–	–	–
Mild	–	10 (63%)	16 (100%)	2 (13%)	–	–	–
Moderate	–	–	–	14 (88%)	9 (56%)	–	–
Severe	–	–	–	–	7 (44%)	16 (100%)	16 (100%)

*WNL* within normal limits

^a^One patient participant’s individual bookmark placement for Upper Extremity Function and Pain Interference was omitted due to illogical order

For Physical Function, although groups were split evenly between a threshold of *T* = 50 and 45 for within normal limits/mild, the majority identified *T* = 50 when working independently. Similarly, groups were evenly divided between *T* = 40 and 35 as the threshold between mild and moderate, but the majority of patients and clinicians identified *T* = 40 when working independently. Three groups identified *T* = 30 as the threshold between moderate and severe. However, the majority identified *T* = 25 when working independently.

For Pain Interference, 3 of 4 groups identified *T* = 50 as the threshold between within normal limits and mild, yet working independently, the majority of patients identified *T* = 55. One patient group was unable to reach consensus on whether the vignette with *T* = 57.5 was mild or moderate, but the majority of participants identified the threshold between mild and moderate at *T* = 60. Groups were evenly divided between *T* = 65 and 70 as the threshold between moderate and severe. Working independently, participants were almost evenly split on what severity label (moderate or severe) should be assigned to the vignette at *T* = 67.5.

To facilitate the use of score thresholds, we make suggestions for score ranges for each PROMIS item bank (see Table 3). In cases where the four groups did not agree on the same threshold but a strong majority of participants selected the same threshold when working independently, we deferred to the individually selected threshold. When individual ratings were more divided, we include two possible thresholds.

**Table 3:** Provisional recommended score thresholds

	WNL	Mild		Moderate		Severe
		Lower limit	Upper limit	Lower limit	Upper limit	
Upper Extremity Function	> 40	30	40	20 or 25	30	< 20 or 25
Physical Function	> 50	40	50	25 or 30	40	< 25 or 30
Pain Interference	> 50 or 55	50 or 55	60	60	65 or 70	> 65 or 70

*WNL* within normal limits

## Discussion

Patients with musculoskeletal injuries and orthopedic clinicians determined score ranges associated with labels describing severity (e.g., mild) by using bookmarking methods with PROMIS Upper Extremity Function, Physical Function, and Pain Interference measures. No significant variation between patient and clinician perspectives emerged. In most cases, the majority opinion as judged by individual ratings prior to group discussion was aligned with the final group consensus. However, this was not always the case. It may be that individuals changed their opinions based on persuasive arguments from others in the group or group dynamics factored into the group’s decision (e.g., desire to be agreeable). It may be beneficial to have individuals make a second private judgment on bookmark placement following discussion rather than pushing the group for consensus. This practice is commonly used when bookmarking is applied in educational settings [12].

Having clear thresholds delineating levels of severity for PROMIS scores facilitates their use for a range of aims. However, in some cases, there was not a clear majority opinion on threshold location. As the vignettes were created at 5-point intervals, it may be that the preferred threshold was not located at the midpoint between these vignettes. Additional bookmarking studies with new vignettes, particularly in the score ranges without consensus (e.g., threshold between moderate and severe pain interference) could build evidence for a specific threshold. Additional studies with patients with more diversity in race, ethnicity, and education can inform the degree to which these provisional recommended thresholds are generalizable.

Thresholds for Pain Interference were consistent with PROMIS’s general interpretation guidance (Pain Interference = 55, 60, 70) [25], thresholds from oncology clinicians (*T* = 50, 60, 70) [15], and rheumatology patients and clinicians (*T* = 50, 60, 65 [patients]/70 [clinicians] [18]; *T* = 51, 61, 68 [20]). Similarly, Physical Function thresholds were consistent with PROMIS’s general interpretation guidance (Physical Function = 45, 40, 30) [25] and with thresholds from oncology patients and clinicians (*T* = 50, 35 [patient]/40 [clinician], 20 [patients]/30 [clinicians]) [14], though varied notably, particularly for better functioning individuals, from one study within rheumatology populations [18]. This is not the case with Upper Extremity. To our knowledge, this is the first bookmarking study with PROMIS Upper Extremity Function. It is notable that this item bank’s range (vignettes 47.5 to 17.5) is different from other PROMIS item banks. The bank is focused on assessing individuals with upper extremity concerns and therefore is less precise for high functioning individuals. Consequently, score interpretation for PROMIS Upper Extremity Function should be tailored specifically to this item bank. General PROMIS *T*-score interpretation may not be appropriate.

Although other bookmarking studies have been conducted in-person, we were able to generate score ranges using a videoconference platform. Many of the components of in-person groups (e.g., use of manipulable paper materials, confidential collection of participants’ initial bookmark placement, moderator’s ability to display materials to all participants, ability for all attendees to see each other’s faces) could be translated to this platform. All participants contributed to the discussion. Although no mode effects were identified between in-person and videoconference in a different PROM-focused study (Time Trade Off interviews), the degree to which mode is an important factor in bookmarking PROMs is unknown [26].

This study has several limitations. First, inherent in qualitative work are small samples of participants. Although efforts were made to include diverse patient and clinician perspectives in the discussions of severity thresholds, the sample is not representative of the full population that experiences or treats musculoskeletal injuries. Our patient participants were primarily white, female, and highly educated. Our small sample size limits our understanding of how specific factors or experiences influence how one perceives level of impairment or dysfunction. Additionally, by conducting bookmarking sessions in a videoconference platform, only those individuals with access to the required technology (e.g., high-speed Internet) and comfort using it were able to participate. At the same time, our virtual adaptation of bookmarking removed other potential barriers for patients who might find in-person participation burdensome.

## Conclusions

Bookmarking methods can identify the ranges of PROMIS scores associated with labels describing the level of severity (e.g., mild). As evidence for specific score thresholds accumulates, we will better understand how factors like diagnosis, treatment, or patient characteristics influence threshold location. These descriptive labels can be used to better interpret the measures when collected in routine clinical care, inform PROMIS-based quality measure development, and facilitate interpretation of research findings. For example, knowing an intervention group’s mean PROMIS Physical Function score is 40 could be translated for patients to understand the expected outcome is on the cusp between mild and moderate dysfunction. Future research is needed to identify if, when, and how labels that describe severity can translate into clinical action for an individual patient.

## Data Availability

Available upon request.
